# Global Lipid Guidelines: More Aligned Than They Appear

**DOI:** 10.5334/gh.1559

**Published:** 2026-05-20

**Authors:** Yashendra Sethi, Kunal Mahajan, Maciej Banach

**Affiliations:** 1 PearResearch, Dehradun, 248001, India; 2 Department of Medicine, Subharti Medical College, Swami Vivekanand Subharti University, Meerut, 250005, India; 3 Department of Cardiology, Himachal Heart Institute, Mandi, Himachal Pradesh, 175021, India; 4 Department of Preventive Cardiology and Lipidology, Medical University of Lodz (MUL), Lodz, Poland; 5 Faculty of Medicine, The John Paul II Catholic University of Lublin, Poland; 6 Liverpool Centre for Cardiovascular Science at University of Liverpool, Liverpool John Moores University and Liverpool Heart & Chest Hospital, Liverpool, United Kingdom

**Keywords:** Lipid guidelines, Global, ESC, ACC, LAI, ILEP

## Abstract

Contemporary lipid guidelines are often portrayed as divergent, creating uncertainty about optimal lipid-lowering strategies in clinical practice. However, major contemporary recommendations—including the 2025 European guidelines, the 2026 American guidelines, the Lipid Association of India (LAI) consensus, and the International Lipid Expert Panel (ILEP) statements—demonstrate substantial conceptual convergence. All endorse earlier identification of high- and extreme-risk phenotypes, intensive lowering of low-density lipoprotein cholesterol (LDL-C) and apolipoprotein B, and prompt use of combination lipid-lowering therapy when monotherapy is unlikely to achieve therapeutic goals. Although differences persist in risk assessment tools, imaging utilization, and implementation frameworks, these largely reflect regional epidemiology, healthcare systems, and affordability rather than fundamental scientific disagreement. Across primary and secondary prevention settings, the unifying paradigm is increasingly “earlier, lower, longer,” emphasizing cumulative lifetime exposure to atherogenic lipoproteins as the principal determinant of atherosclerotic cardiovascular disease risk. The major challenge is therefore no longer guideline discordance, but translating convergent evidence into systematic, timely, and sustained implementation in routine care.

## Graphical abstract

**Figure:**
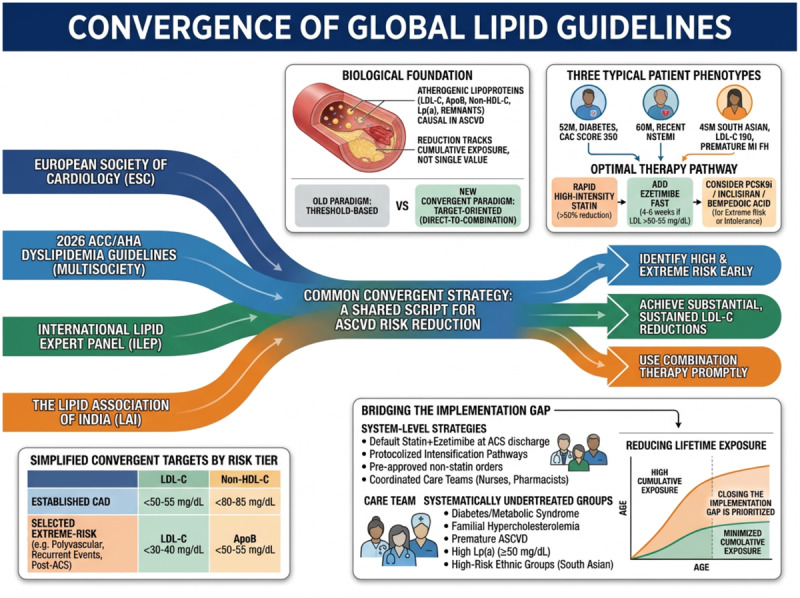


The contemporary lipid guidelines are often portrayed as competing, leaving clinicians unsure which one to follow at the bedside. In reality, all major guidelines now function as a family of closely related strategies: the European Society of Cardiology (ESC) guidelines, the Lipid Association of India (LAI) guidelines, the International Lipid Expert Panel (ILEP) guidelines, and the 2026 American College of Cardiology/American Heart Association (ACC/AHA) dyslipidemia guidelines—issued on behalf of 11 cardiovascular and metabolic societies—all point in the same direction (Table 1) (1,2,3,4,5). The shared message is to identify high- and extreme-risk patients early, achieve substantial and sustained reductions in low-density lipoprotein cholesterol (LDL-C) and apolipoprotein B (apoB), and use combination therapy promptly whenever a single agent is unlikely to be enough.

**Table 1:** Comparative Framework: ESC/EAS 2025 vs ACC/AHA 2026 vs LAI 2023 vs ILEP 2024/2025.


DOMAIN	ESC/EAS 2025	ACC/AHA 2026	LAI (INDIA) 2023	ILEP (2024/2025)	CLINICAL INTERPRETATION

**Underlying Philosophy**	Target-driven, risk-tiered LDL lowering	Personalized risk estimation with a goal-based hybrid model	Early aggressive approach reflecting high baseline risk	‘Earlier, lower, longer’ + upfront intensive therapy	Unified causal paradigm of LDL exposure across the lifespan

**Risk Assessment Tool**	SCORE2/SCORE2-OP	PREVENT (10- & 30-year risk)	LAI ASCVD Risk Calculator	Risk-based + emphasis on clinical risk dominance over scores in high-risk states	Different tools, shared goal: lifetime risk identification

**Risk Categories**	Five tiers: Low → Extreme	Four tiers: Borderline → Very High	Five tiers incl. Extreme (A/B/C subclassification)	Expanded very high & extreme risk definitions	Variation reflects epidemiology, not disagreement

**Definition of Very High Risk**	ASCVD, CKD, DM with TOD, SCORE2 ≥20%	≥2 ASCVD events OR one event + high-risk features	ASCVD, DM with complications, HeFH	ASCVD + high residual risk; includes post-ACS/stroke emphasis	Broad alignment across frameworks

**Extreme Risk Category**	Recurrent ASCVD or polyvascular disease	Not formally defined	Strongly emphasized; subclassified (A/B/C)	Strongly emphasized; includes ACS, stroke, polyvascular disease, FH	Consolidates ultra-high-risk phenotype

**LDL-C Targets (Very High Risk)**	<55 mg/dL + ≥50% reduction	<55–70 mg/dL + individualized	<50 mg/dL	<55 mg/dL with rapid attainment via combination therapy	Converging targets with differing urgency

**LDL-C Targets (Extreme Risk)**	<40 mg/dL	Not defined	<50 mg/dL; optional <30 mg/dL (A/B); up to 10–15 mg/dl in Category C	<40 mg/dL; consider <30 mg/dL in selected patients	Emergence of ultra-low LDL paradigm

**Role of CAC**	Risk modifier	Direct determinant of therapy	Risk-enhancing feature	Secondary to immediate treatment in high-risk states	CAC shifting from refinement → trigger

**CAC-Based Targets**	Not target-defining	CAC-guided LDL thresholds	CAC-driven intensification	Less emphasized vs clinical risk urgency	Imaging complements—not replaces—clinical risk

**Imaging Beyond CAC**	Limited	Increasing (CT-based)	Strong (carotid/femoral plaque)	Adjunctive; not delaying therapy	Imaging is evolving, but not central in acute risk

**Non-HDL-C Role**	Secondary target	Co-primary target	Co-primary target	Co-equal to LDL-C in high TG/metabolic states	Reflects total atherogenic burden

**ApoB Role**	Recommended in discordance	Refines risk	Integrated in high-risk	Preferred marker of atherogenic particle burden	ApoB gaining primacy

**Lp(a)**	Risk enhancer	Universal one-time measurement	Strong emphasis	Central residual risk factor; strong emphasis	Universal agreement on causal role

**Therapeutic Strategy**	Stepwise escalation	Flexible sequencing	Early combination therapy	Upfront combination therapy (double/triple) in high-risk	Shift from escalation → early intensification

**Primary Prevention**	Structured, risk-based	Earlier intervention (≥3–5% risk)	Lower thresholds	Early intervention guided by lifetime exposure	Movement toward earlier treatment

**Secondary Prevention (ACS/Stroke)**	Early statin + ezetimibe	Flexible early intensification	Immediate aggressive combination	Immediate upfront combination therapy mandatory	‘Treat fast, treat deep’ paradigm

**Severe Hypercholesterolemia (LDL ≥190)**	High intensity + add-ons	Early add-on therapy	Aggressive multi-drug	Immediate combination ± PCSK9/Inclisiran	No delay strategy across frameworks

**Triglyceride Approach**	Secondary focus	Integrated	Strong emphasis	Residual risk focus (TRLs, remnant cholesterol)	TG-rich lipoproteins increasingly relevant

**Residual Risk Concept**	Expanding beyond LDL	ApoB, Lp(a), inflammation	Strong metabolic focus	Multidimensional (ApoB, Lp(a), TG, inflammation)	Shift to a multi-marker paradigm

**Time-to-Target Philosophy**	Stepwise, structured	Flexible	Early aggressive	Immediate goal attainment (weeks, not months)	Same endpoint, different speed

**Population Context**	Aging European cohorts	Heterogeneous population	Premature ASCVD in South Asians	Global, high-risk focus (ACS/stroke/FH)	Epidemiology shapes strategy

**Implementation Style**	Algorithmic, system-driven	Shared decision-making	Prevention-oriented	Outcome-driven, intensity-focused	Differences reflect systems, not science


Abbreviations: ACC/AHA = American College of Cardiology/American Heart Association; ACS = acute coronary syndrome; ASCVD = atherosclerotic cardiovascular disease; ApoB = apolipoprotein B; CAC = coronary artery calcium; CKD = chronic kidney disease; CT = computed tomography; DM = diabetes mellitus; EAS = European Atherosclerosis Society; ESC = European Society of Cardiology; FH = familial hypercholesterolemia; HeFH = heterozygous familial hypercholesterolemia; HDL-C = high-density lipoprotein cholesterol; ILEP = International Lipid Expert Panel; LAI = Lipid Association of India; LDL-C = low-density lipoprotein cholesterol; Lp(a) = lipoprotein(a); TG = triglycerides; TRLs = triglyceride-rich lipoproteins.

The biological foundation for this convergence is now difficult to dispute. Atherogenic lipoproteins—LDL-C, non-HDL-C, apolipoprotein B-containing particles, triglyceride-rich remnants, and lipoprotein(a) (Lp(a))—are causal in atherosclerotic cardiovascular disease (ASCVD), and event reduction tracks cumulative exposure rather than any single LDL-C value (6). Different frameworks package this in different languages, but they all translate into the same clinical principle: treat earlier in the risk trajectory, lower LDL-C more aggressively as risk rises, and then keep levels low over the long term instead of tolerating oscillation around a threshold.

Three clinic scenarios that every cardiologist and internist recognizes make this concrete: a 52-year-old with diabetes, metabolic syndrome, and a coronary artery calcium (CAC) score of 350; a 60-year-old with recent non-ST-elevation myocardial infarction; and a 45-year-old South Asian man with LDL-C of 190 mg/dL and a family history of premature myocardial infarction. Across the ESC/EAS, ACC/AHA, LAI, and ILEP recommendations, all three warrant rapid initiation of high-intensity statin therapy targeting at least a 50% LDL-C reduction, with a prespecified plan to add ezetimibe within weeks if LDL-C remains above roughly 50–55 mg/dL, and to consider a monoclonal proprotein convertase subtilisin/kexin type 9 (PCSK9) inhibitor or inclisiran (a small interfering RNA targeting hepatic PCSK9 synthesis), or bempedoic acid in those with extreme risk or statin intolerance (1,2,3,4,5). For these phenotypes, a prolonged ‘statin-monotherapy-first’ phase is no longer defended by any major guidelines; what differs is only how explicitly each document says ‘start with combination’ versus ‘add promptly when needed’.

Historically, clinicians were taught to choose between a target-based European approach and a threshold-based US approach (7). With recent US guidance re-introducing explicit LDL-C and non-HDL-C goals in high-risk groups, that dichotomy has largely dissolved. A more useful framing at the bedside is deliberately simple: set a reasonable LDL-C (and non-HDL-C or apoB) goal based on vascular risk, measure the response promptly, and intensify therapy preferably by adding rather than substituting agents until the patient is there (8). In established coronary artery disease, this generally translates to LDL-C <50–55 mg/dL and non-HDL-C <80–85 mg/dL, with selected extreme-risk patients who either have polyvascular ASCVD, are recurrent-event patients, or have previously had an acute coronary syndrome (ACS) or other concurrent severe conditions allocated a target of <30–40 mg/dL LDL-C and apoB <50–55 mg/dL when feasible. This ‘rule of thumb’ is compatible with all major guidelines and easy to communicate (1,2,3,4,5).

Risk equations, CAC scoring, carotid or femoral plaque imaging, and newer lifetime risk tools can give the impression that guidelines are diverging, yet their clinical use converges on one purpose: to move borderline or ‘uncertain’ patients into or out of pharmacologic therapy and to justify more ambitious lipid goals when the atherosclerotic substrate is clearly present. In a 45-year-old without prior events but with risk enhancers, a CAC score of 0 may justify deferring statin therapy with continued lifestyle and repeat assessment, whereas a CAC score ≥100, carotid plaque, high apoB, remnant cholesterol, or Lp(a) ≥50 mg/dL should prompt earlier statin initiation and more aggressive LDL-C and non-HDL-C lowering, often from the outset as combination therapy rather than staged intensification (1,2,3,4,5).

Despite converging guidance, registry and real-world data show that most high- and very-high-risk patients still fail to reach even conservative LDL-C thresholds, and attainment of 55 or 40 mg/dL remains uncommon (7). Closing this implementation gap would be more impactful than reconciling minor textual differences between documents. Practical, system-level strategies include defaulting to high-intensity statins plus ezetimibe (ideally as a fixed-dose combination) at discharge in ACS and polyvascular patients; scheduling follow-up lipid testing within 4–6 weeks of any therapy change; embedding pre-approved non-statin add-on orders for very-high- and extreme-risk phenotypes; and empowering nurses, pharmacists, and coordinated care teams to run protocolized intensification pathways rather than relying on sporadic visits (1,2,3,4,5).

Documents written for different populations also converge in highlighting systematically undertreated groups: patients with diabetes and metabolic syndrome, familial hypercholesterolemia, premature ASCVD, high Lp(a), and from high-risk ethnic groups such as South Asians. In practice, this should prompt a shift from a uniform ‘treat when 10-year risk is high enough’ paradigm toward proactively recognizing these phenotypes as intrinsically high- or extreme-risk and aiming for lower LDL-C, non-HDL-C, and apoB targets; faster intensification; and earlier use of combination therapy than might otherwise seem intuitive. In resource-constrained settings, this means ensuring universal access to high-intensity statins and generic ezetimibe as the base and reserving injectable therapies and icosapent ethyl for those with the highest residual risk or recurrent events.

Residual differences between guidelines mainly reflect context—epidemiology, health systems, and affordability—rather than true disagreement. European documents emphasize structure and an ‘extreme-risk’ tier; US multisociety guidance stresses flexibility and imaging; Indian guidance underscores earlier disease; ILEP turns these themes into algorithms favouring upfront dual or triple therapy in those with the greatest residual risk (1,2,3,4,5). For clinicians, these are variations on a shared script: identify high- to extreme-risk patients early, start high-intensity statins promptly (preferably with ezetimibe in early stages), define and communicate clear LDL-C and non-HDL-C/apoB goals, remeasure within weeks, and escalate with combination therapy when goals are missed or baseline risk is extreme. If this common core is implemented reliably, which specific guideline is cited matters less than ensuring each eligible patient spends as little of their life as possible exposed to high concentrations of atherogenic lipoproteins.

## Financial interests

Dr. Banach: Speaker’s bureau: Amgen, Adamed, Daiichi Sankyo, KRKA, MSD, Polpharma, Mylan/Viatris, Novartis, Novo-Nordisk, Pfizer, Sanofi, Teva, Zentiva; consultant to Adamed, Amgen, Daiichi Sankyo, Lilly, MSD, New Amsterdam, Novartis, Novo-Nordisk, Sanofi, Teva; Grants from Amgen, Daiichi Sankyo, Viatris, Sanofi, and Teva. All other authors declare they have no financial interests.

## References

[1] Mach F, Koskinas KC, Roeters Van Lennep JE, et al. 2025 Focused Update of the 2019 ESC/EAS Guidelines for the management of dyslipidaemias. Eur Heart J. 2025;46(42):4359–4378. DOI: 10.1093/eurheartj/ehaf190 40878289

[2] Puri R, Bansal M, Mehta V, et al. Lipid Association of India 2023 update on cardiovascular risk assessment and lipid management in Indian patients: Consensus statement IV. J Clin Lipidol. 2024;18(3):e351–e373. DOI: 10.1016/j.jacl.2024.01.006 38485619

[3] Banach M, Reiner Ž, Surma S, et al. 2024 Recommendations on the Optimal Use of Lipid-Lowering Therapy in Established Atherosclerotic Cardiovascular Disease and Following Acute Coronary Syndromes: A Position Paper of the International Lipid Expert Panel (ILEP). Drugs. 2024;84(12):1541–1577. DOI: 10.1007/s40265-024-02105-5 39497020 PMC11652584

[4] Banach M, Toth PP, Ahn HJ, Bielecka-Dabrowa A, Cicero AFG, Covic A, et al. Lipid management for primary and secondary stroke prevention consensus paper of the International Lipid Expert Panel (ILEP). Prog Cardiovasc Dis. 2026 Jan–Feb;94:78–110. DOI: 10.1016/j.pcad.2025.11.003 41249078

[5] Blumenthal RS, Morris PB, Gaudino M, et al. 2026 ACC/AHA/AACVPR/ABC/ACPM/ADA/AGS/APhA/ASPC/NLA/PCNA Guideline on the Management of Dyslipidemia. JACC. Published online March 2026:S0735109725102544. DOI: 10.1016/j.jacc.2025.11.016

[6] Lee YJ, Lee SJ, Kim JW, et al. Intensive LDL Cholesterol Targeting in Atherosclerotic Cardiovascular Disease. N Engl J Med. Published online March 28, 2026. DOI: 10.1056/NEJMoa2600283 41910315

[7] Pradhan A, Thandi P, Mahajan K, Iellamo F, Perrone-Filardi P, Perrone MA. 2025 ESC/EAS Dyslipidemia Guidelines Focused Update: Intensifying Prevention, Risk Stratification, and Therapy. Am J Cardiol. Published online March 3, 2026:S0002-9149(26)00109-8. DOI: 10.1016/j.amjcard.2026.02.059 41785983

[8] Mahajan K, Jena S, Dutta D, et al. Efficacy and Safety of Upfront Oral Triple Lipid-Lowering Therapy: A Systematic Review and Single-Arm Meta-Analysis. Diabetes Obes Metab. Published online March 16, 2026. DOI: 10.1111/dom.70663 41834762

